# Transition-state aromaticity and its relationship with reactivity in pericyclic reactions

**DOI:** 10.3762/bjoc.21.125

**Published:** 2025-08-12

**Authors:** Israel Fernández

**Affiliations:** 1 Departamento de Química Orgánica, Facultad de Ciencias Químicas, Universidad Complutense de Madrid, 28040-Madrid, Spain

**Keywords:** activation barrier, activation strain model, aromaticity, computational chemistry, transition state

## Abstract

The influence of transition-state aromaticity on the barrier heights of concerted pericyclic reactions is summarized herein. To this end, selected representative examples ranging from fundamental processes such as Diels–Alder or Alder–ene reactions to double-group transfer reactions or 1,3-dipolar cycloadditions involving metal complexes are presented. It is found that while more synchronous processes tend to exhibit greater aromatic character in their transition states, this increased aromaticity does not necessarily correlate with lower activation barriers. State-of-the-art computational methods on reactivity, such as the combined activation strain model (ASM)–energy decomposition analysis (EDA) method, reveal that factors other than aromaticity govern the barrier heights of these pericyclic reactions.

## Introduction

Aromaticity is arguably one of the most fundamental and extensively studied concepts in chemistry [1–3]. Initially introduced to account for the remarkable stability, low reactivity, and unique structural features of benzene and related aromatic hydrocarbons, the concept has undergone a significant evolution [4]. Since the introduction of the famous Hückel’s (4*n* + 2) rule [5], first clearly defined by Doering and Detert [6], this concept has been extended well beyond benzenoid molecules to organometallic, inorganic, and even saturated molecules. As a result, besides classical π-aromaticity, other aromaticity types such as Möebius, spherical, or excited-state aromaticity (to name a few) have been introduced [7–11].

**Scheme 1:**
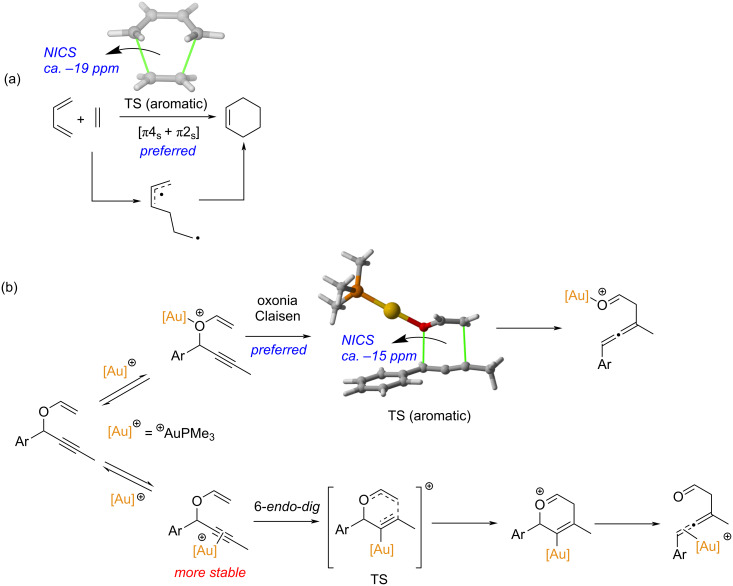
(a) Diels–Alder cycloaddition reaction between butadiene and ethylene. (b) Gold(I)-catalyzed propargyl rearrangement.

The concept of aromaticity was also extended to the transition states (TSs) of concerted pericyclic reactions as early as 1938 when Evans and Warhurst [12] recognized the relationship between the six π-electrons of benzene and the six delocalized electrons in the transition structure of the Diels–Alder cycloaddition reaction between butadiene and ethylene “*…Very qualitatively, we may say that whereas in the initial state the mobile electrons are those characteristics of an ethylene and a butadiene structure, in the TS they simulate the behavior of a benzene molecule*”. This initial recognition was later generalized by Zimmerman and Dewar [13–14] who extended the Hückel–Möbius aromaticity concepts to different pericyclic reactions including not only cycloaddition reactions but also electrocyclizations or sigmatropic reactions, linking them to the Woodward–Hoffmann rules [15]. In this regard, it has been suggested that thermally allowed pericyclic reactions take place preferentially through concerted aromatic transition states, which are favored energetically and therefore display faster rates (i.e., lower barriers) [16–17]. This is in line with the seminal conclusion by Evans in 1939 [18] indicating that “...*the lowering of the activation energy arises from the increased mobility which the π electrons of such reactions possess in the TS*.” Indeed, it was found, both experimentally and computationally, that the concerted pathway (i.e., involving an aromatic TS) of the butadiene + ethylene reaction proceeds with a lower barrier (up to 7 kcal/mol) than the corresponding stepwise pathway leading to a diradical intermediate (Scheme 1a) [19–20]. Similar preferences for concerted pathways over stepwise mechanisms were also found for dipolar cycloadditions and Cope rearrangements [20]. More recently, Alabugin and co-workers reported that the gold(I)-catalyzed propargyl rearrangement depicted in Scheme 1b also follows a concerted oxonia Claisen pathway (via an aromatic TS) rather than through a higher barrier 6-*endo*-*dig* cyclization [21], which provides further support to the barrier-lowering effect induced by the aromaticity of the transition state.

While the purported ‘‘aromatic stabilization’’ is mainly established based on comparisons to transition states of alternative stepwise reaction routes, its extension to highly related processes following concerted mechanisms has not been considered. For instance, very little is known about the influence of TS-aromaticity (if any) on the barrier heights of catalyzed pericyclic reactions and related transformations. Our group has been focused on this aspect in recent years by exploring the interplay between the aromaticity of the transition state and the activation barriers of different pericyclic reactions spanning from the parent Diels–Alder reactions to higher-order cycloadditions or even transition-metal-mediated transformations [22–29]. By selecting illustrative examples, in this perspective article, we shall show the relationship between transition-state aromaticity and reactivity in representative pericyclic reactions.

## Perspective

### Lewis acid-catalyzed Diels–Alder cycloadditions

It is well-established that catalytic amounts of a Lewis acid (LA) can significantly accelerate Diels–Alder cycloaddition reactions [30–31]. In addition, these LA-catalyzed cycloadditions not only exhibit higher reaction rates but also become, in many cases, more regio- and stereoselective than the corresponding uncatalyzed reactions. For these reasons, LAs have been (and still are) widely used in the synthesis of a good number of target molecules, including complex natural products [32–35].

Typically, the LA binds the dienophile through a donor–acceptor interaction (usually involving a carbonyl group) which results in a significant stabilization of the dienophile’s LUMO. As a consequence, the corresponding HOMO(diene)–LUMO(dienophile) energy gap becomes smaller, which, according to the frontier molecular orbital (FMO) theory, constitutes the origin of the observed acceleration (following the so-called *LUMO-lowering concept* in catalysis) [36–39]. This widely accepted rationalization of the effect of the LA in the Diels–Alder reaction therefore does not consider a possible influence of the aromaticity of the transition state on the barrier heights. For this reason, and to understand the ultimate origin of the faster rates observed in the LA-catalyzed Diels–Alder reactions, we focused on the textbook reaction involving isoprene and (*s-trans*) methyl acrylate catalyzed by different LAs [40].

Our results confirm that the LAs significantly accelerate the cycloaddition reaction, which in all cases proceeds in a concerted manner (Table 1). The computed barriers follow the same trend as the relative Lewis acidity of the catalyst (measured by the Child’s method [41–43]), but do not follow the same trend as the energy of the LUMO(dienophile). This finding therefore challenges the traditionally used LUMO-lowering concept as the ultimate factor controlling the catalysis in these Diels–Alder reactions.

**Table 1:** LA-catalyzed Diels–Alder reactions between isoprene and methyl acrylate.

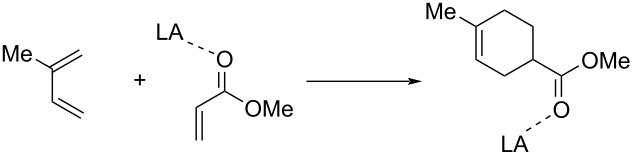			

LA	Δ*E*^≠^ [kcal mol^−1^]^a^	Relative Lewis acidity^b^	ε_LUMO_ (dienophile) [eV]

none	16.2		−2.6
SnCl_4_	12.3	0.52 ± 0.04	−3.7
TiCl_4_	11.8	0.66 ± 0.03	−4.0
ZnCl_2_	10.8		−4.3
BF_3_	10.0	0.77 ± 0.02	−3.8
AlCl_3_	7.6	0.82	−4.2

^a^Computed activation barriers at the (TightPNO)DLPNO-CCSD-(T) /CBS(3,4/def2)//ZORA-BP86/TZ2P level; ^b^relative Lewis-acidity scale based on Δδ-values of H3 resonances of various bases related to methyl crotonate, data taken from reference [41].

**Figure 1:**
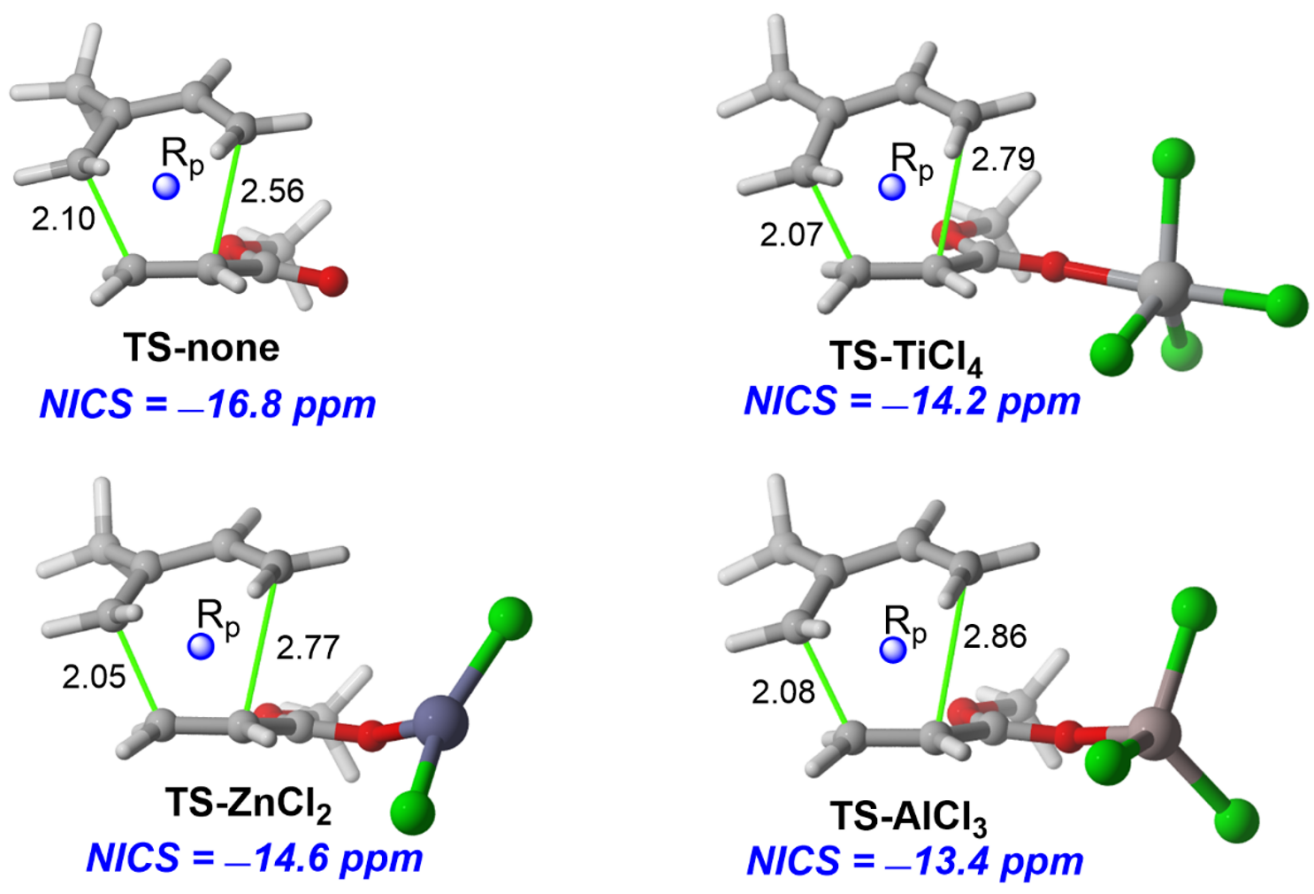
Transition states computed for the Diels–Alder cycloaddition reaction between isoprene and methyl acrylate catalyzed by Lewis acids. Bond distances are given in angstroms and NICS(3, +1) values in ppm.

We then explored whether the aromaticity of the involved transition states plays a role in the observed reduction of the barriers. To this end, we computed the nuclear independent chemical shift (NICS) [44] values at the (3, +1) ring critical point of the electron density of the TSs. This point was recommended due to its high sensitivity to diamagnetic effects and its unambiguous character [45–46], which is crucial for the considered transition structures due to their highly asynchronous nature. As shown in Figure 1, all the TSs can be considered aromatic in view of their negative NICS(3, +1) values. Interestingly, the TS-aromaticity of all species is not only comparable (ca. −15 ppm), but slightly decreases from the uncatalyzed reaction to the analogous AlCl_3_-catalyzed cycloaddition. If we take into account that aromaticity is usually associated with bond-length equalization [2–3], this finding is not surprising as the uncatalyzed reaction is clearly more synchronous than their catalyzed counterparts (see bond lengths in Figure 1). Therefore, it becomes evident that the trend in the TS-aromaticity is opposite to the reactivity trend, which suggests that aromaticity is not behind the observed acceleration in the catalyzed reactions.

**Figure 2:**
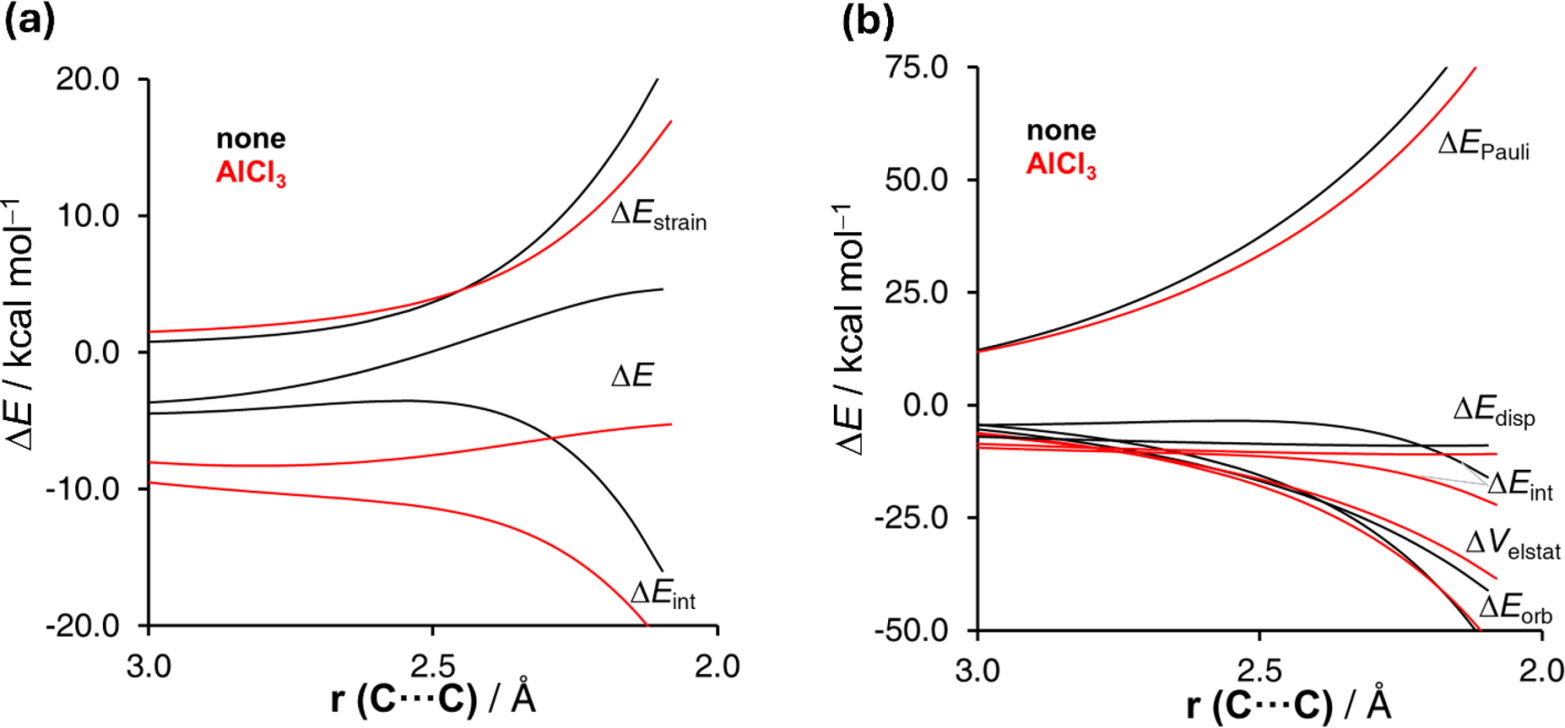
Comparative activation strain analyses (a) and energy decomposition analysis (b) of the Diels–Alder cycloaddition reaction between isoprene and methyl acrylate (black lines) and the analogous process catalyzed by AlCl_3_ (red lines) and projected onto the C···C bond-forming distance.

Once we found that neither aromaticity nor favorable orbital (HOMO(diene)–LUMO(dienophile)) interactions are responsible for the faster rates exhibited by the catalyzed reactions, we applied the activation strain model (ASM) of reactivity [47–48] to quantitatively understand the origin of the acceleration in these pericyclic reactions. This model decomposes the electronic energy (Δ*E*) into two terms, namely the strain (Δ*E*_strain_) that results from the distortion of the individual reactants and the interaction (Δ*E*_int_) between the deformed reactants along the reaction coordinate, defined in this case by the shortest C···C bond-forming distance. From the data in Figure 2a, which shows the computed activation strain diagrams (ASDs) for the uncatalyzed and AlCl_3_-catalyzed reactions from the beginning of the process up to the respective TSs, it becomes clear that the lower barrier computed for the catalyzed cycloaddition is due to a lower strain (which derives from the higher asynchronicity of the process) and, mainly, to a much stronger interaction between the deformed reactants along the entire reaction coordinate. According to the energy decomposition analysis (EDA) method [49–50], which further decomposes the crucial Δ*E*_int_ term into three physically meaningful energy terms, namely the classical electrostatic interaction (Δ*V*_elstat_), the Pauli repulsion (Δ*E*_Pauli_) arising from the repulsion between occupied closed-shell orbitals of both deformed reactants, and the orbital interaction (Δ*E*_orb_) that accounts for charge transfer and polarization, the stronger interaction computed for the AlCl_3_-catalyzed does not result from stronger orbital interactions (as traditionally viewed) but exclusively from a less destabilizing Pauli repulsion between the deformed reactants (Figure 2b).

The less destabilizing Pauli repulsion computed for the catalyzed cycloaddition directly originates from the reduction of the electron density at the reactive C–C double bond of the dienophile, which translates into a lower <π(diene)|π(dienophile)> molecular orbital overlap (*S* = 0.13 vs *S* = 0.07 for the uncatalyzed and catalyzed reactions, respectively). In other words, there occurs a significant polarization of the conjugated π-system away from the C–C double bond when the LA binds the carbonylic oxygen of methyl acrylate, which results in a less destabilizing 4-electron interaction with the diene and ultimately, in a lower activation barrier. The term “Pauli-repulsion lowering in catalysis” [51] was coined to describe this effect, which has been proven to be general and applicable not only to related LA-catalyzed pericyclic reactions (as shown in the following subchapter) but also to other processes such as aza-Michael additions or processes catalyzed by species able to establish noncovalent interactions with the reactants [52–57].

### Lewis acid-catalyzed carbonyl–ene reactions

Similar to the Diels–Alder cycloaddition reaction, the Alder–ene reaction [58–59] constitutes a fundamental reaction in organic chemistry that has been applied to the synthesis of a number of target molecules owing to its remarkable functional group tolerance [60–61]. Despite that, the main shortcoming of this transformation is its relatively high barrier, which is translated into high reaction temperatures that severely limit the scope of the process. Nevertheless, as Diels–Alder cycloadditions, Alder–ene reactions can be efficiently accelerated (i.e., having lower barriers) upon the addition of catalytic amounts of a Lewis acid [62], which again, typically binds a carbonyl group in the enophile partner. In this sense, the acceleration is even more significant in the so-called carbonyl–ene reaction [63], an analogous process where a carbonyl group directly acts as the enophile. This transformation has also been applied to the synthesis of complex natural products such as (+)-steenkrotin A [64] or (±)-andrastin C [65], among others.

In order to understand the factors leading to the observed reactivity enhancement, we first compared the parent carbonyl–ene reaction between 1-butene and acetaldehyde with the analogous processes catalyzed by different LAs [66] (Table 2). As expected, we found that the reduction of the activation barrier (up to ca. 25 kcal/mol) directly correlates with the relative Lewis acidity of the catalyst. In addition, the process becomes more and more asynchronous as the acidity of the catalyst increases, which strongly resembles the trends found for Diels–Alder reactions discussed above.

**Table 2:** Computed free activation energies (Δ*G*^≠^, in kcal/mol), synchronicity (*S*_y_), and NICS(3, +1) values in the corresponding TSs (in ppm) of the considered carbonyl–ene reactions.

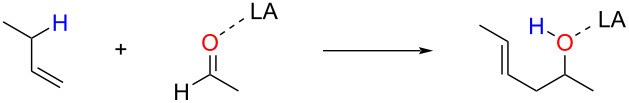				

Catalyst	Δ*G*^≠^ [kcal mol^−1^]	*S*_y_^a^	NICS(3, +1) [ppm]	Lewis acidity^b^

none	44.1	0.87	−19.9	
BMe_3_	34.8	0.62	−13.5	
BPh_3_	28.0	0.58	−11.2	
SnCl_4_	27.4	0.61	−9.9	0.52 ± 0.04
AlMe_2_Cl	25.8	0.59	−10.3	0.59 ± 0.03^c^
InCl_3_	24.7	0.61	−10.5	
TiCl_4_	24.0	0.55	−6.5	0.66 ± 0.03
BF_3_	22.4	0.58	−8.8	0.77 ± 0.02
AlCl_3_	19.3	0.56	−7.8	0.82

^a^*S*_y_ stands for the computed synchronicity (for a perfectly synchronous process, *S*_y_ = 1). All data were computed at the PCM(dichloromethane)-ωB97xD/def2-TZVPP//PCM-(dichloromethane)-ωB97xD/def2-SVP level; ^b^relative Lewis-acidity scale based on Δδ-values of H3 resonances of various bases related to methyl crotonate, data taken from reference [41]; ^c^value for AlEt_2_Cl.

**Figure 3:**
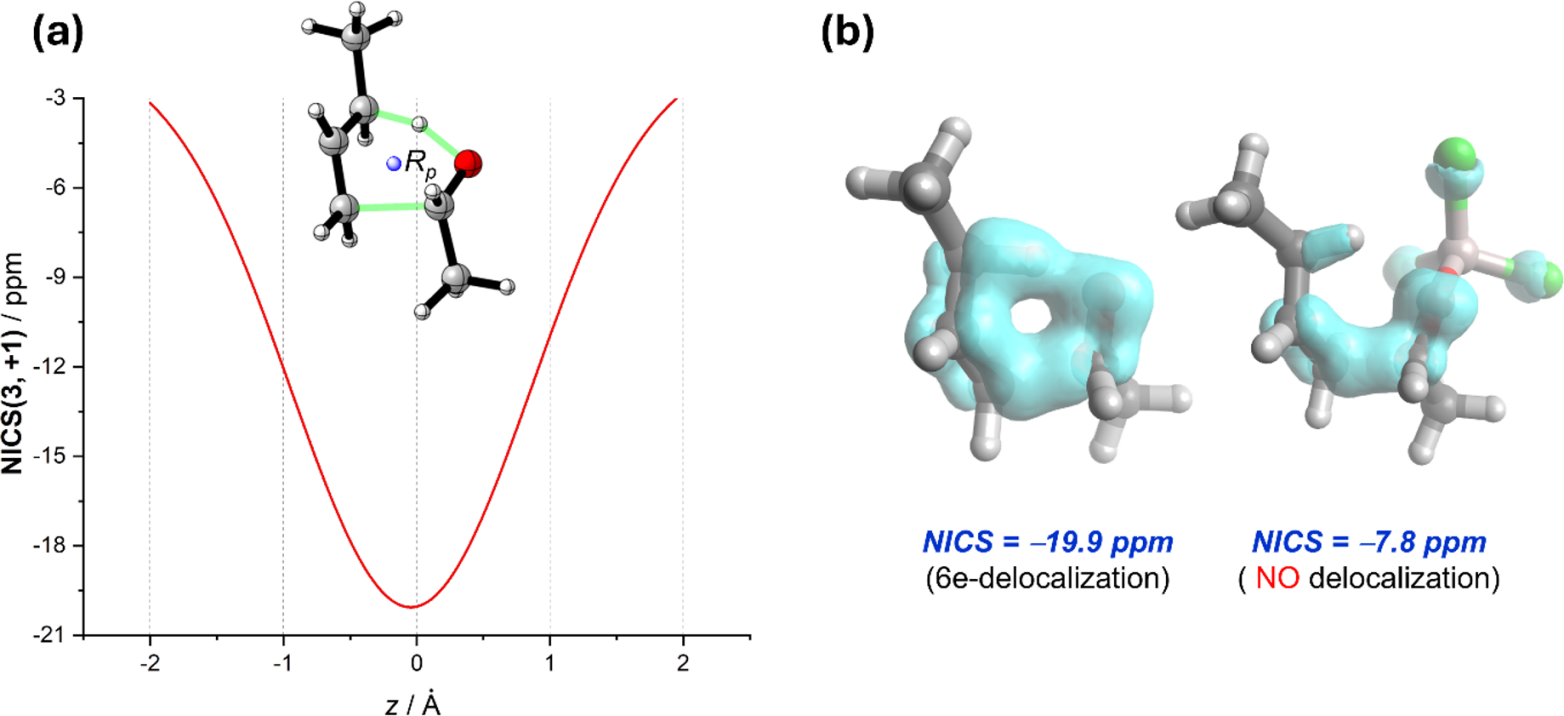
(a) Evolution of the NICS(3, +1) values along a *z*-axis perpendicular to the molecular plane of the TS involved in the 1-butene and acetaldehyde reaction. (b) EDDB plots and NICS(3, +1) values for the carbonyl–ene reaction between 1-butene and acetaldehyde (left) and the analogous process catalyzed by AlCl_3_ (right).

For the parent Alder–ene reaction, which involves an alkene as the enophile, we previously reported that the corresponding six-membered transition state can be considered as in-plane aromatic in view of its highly negative NICS(3, +1) value (ca. −20 ppm) [67]. Not surprisingly, the analogous, uncatalyzed carbonyl–ene reaction features a similar TS-aromaticity as confirmed by the evolution of the NICS values along a *z*-axis perpendicular to the molecular plane which exhibits the bell-shape expected for an in-plane aromatic molecule [45–46] (Figure 3a). Similar to the LA-catalyzed Diels–Alder reactions, the asynchronicity induced by the catalysts results in a reduced TS-aromaticity, which results from the reduction of the six-electron delocalization in the uncatalyzed reaction, as confirmed by the electron density of delocalized bonds (EDDB) method [68] (Figure 3b). Indeed, the trend in the computed NICS(3, +1) values is opposite to the trend in the reactivity (see Table 2), which once again indicates that the TS-aromaticity has practically a negligible influence on the barriers of these pericyclic reactions.

**Figure 4:**
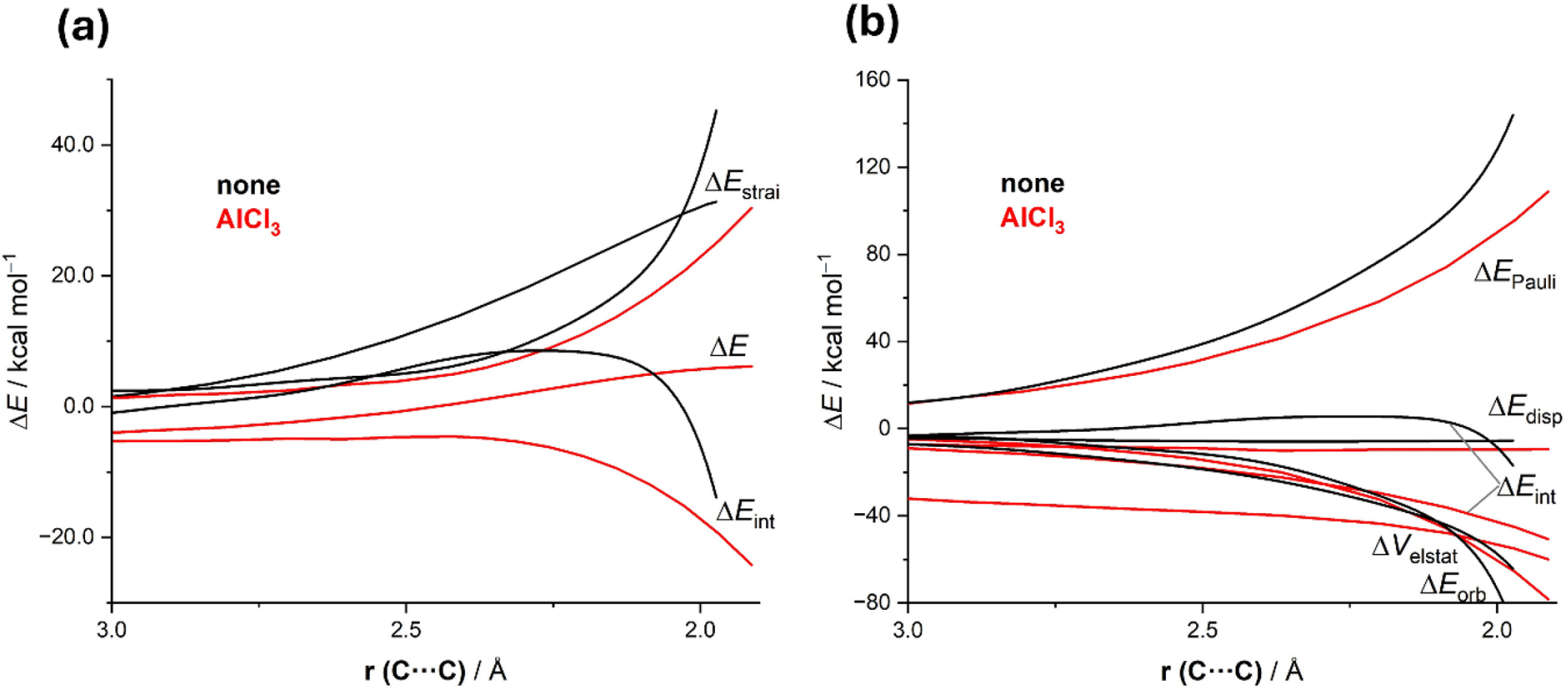
Comparative activation strain analyses (a) and energy decomposition analysis (b) of the carbonyl–ene reaction between 1-butene and acetaldehyde (black lines) and the analogous process catalyzed by AlCl_3_ (red lines) and projected onto the C···C bond-forming distance.

We also applied the combined ASM-EDA approach to gain quantitative insight into the ultimate factors controlling the catalysis in this particular reaction. Once again, the ASM method indicates that the catalyzed process benefits from both a less destabilizing strain (due to the higher asynchronicity) and, to a greater extent, from a much stronger interaction between the deformed reactants along the entire reaction coordinate (see Figure 4a for the extreme situations represented by the uncatalyzed and AlCl_3_-catalyzed reactions). According to the EDA method (Figure 4b), this is almost exclusively due to a significant reduction of the Pauli repulsion between the reactants in the catalyzed reaction. Therefore, once again we found that the LA catalyst induces a significant polarization in the reactive carbonyl group which (i) renders the process more asynchronous and therefore, less aromatic, but (ii) diminishes the destabilizing 4-electron interaction with the C–C double bond of the alkene partner, dramatically reducing the barrier of the process.

### Other non-catalyzed pericyclic reactions

#### Double group transfer reactions

Double group transfer reactions (DGTRs) are a particular type of pericyclic reaction involving the simultaneous migration of two atoms or groups from one compound to another, very often in a concerted manner [69]. Textbook reactions such as the diimide reduction of unsaturated bonds [70–72], the Meerwein–Ponndorf–Verley reduction of carbonyl groups [73] and some type II dyotropic reactions [74–75] constitute representative DGTRs.

**Figure 5:**
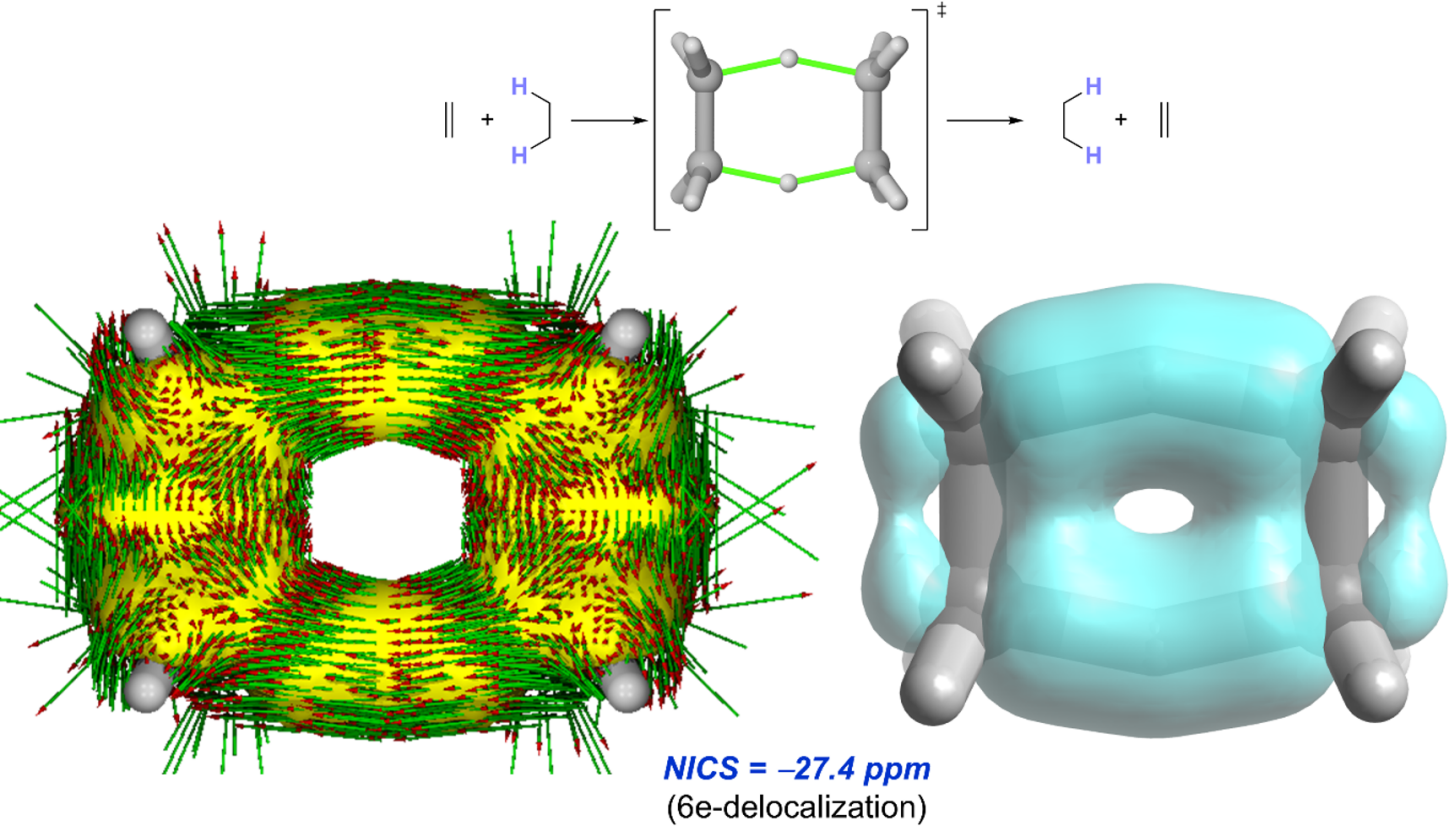
AICD (a) and EDDB (b) plots for the transition state involved in the DGRT between ethene and ethane.

In general, concerted DGRTs are thermally allowed [σ2s + σ2s + π2s] transformations according to the Woodward–Hoffmann rules [15], and therefore proceed through a highly synchronous six-membered transition state. Not surprisingly and similar to the ene reactions discussed above, the delocalization of the involved six electrons within the molecular plane makes the corresponding DGTR transition state in-plane aromatic [76–77]. This is again confirmed not only by the highly negative NICS value computed at the (3, +1) ring critical point but also by the diatropic (i.e., aromatic) ring current observed upon applying the anisotropy of the induced current density (ACID) method [78] or the delocalization observed with the EDDB method (see Figure 5 for the parent process involving the reaction between ethane and ethene).

Typically, DGRTs involving C–H bonds are associated with high barriers (>40 kcal/mol). However, we found that the Meerwein–Ponndorf–Verley-type reaction involving the double hydrogen migration from CH_3_–OH to O=CH_2_ proceeds with a much lower activation barrier of ca. 25 kcal/mol [79]. Interestingly, although the analogous reaction involving CH_3_–CH_3_ and O=CH_2_ proceeds with a much higher barrier, both transformations feature transition states whose in-plane aromaticity is nearly identical according to their NICS(3, +1) values (−24.5 ppm vs −27.2 ppm, respectively). Therefore, it is found once again that there is no clear relationship between the TS-aromaticity and the barrier heights.

The origin of the computed decrease of the activation barrier in the CH_3_–OH to O=CH_2_ as compared to the analogous reaction involving ethane as hydrogen donor can be initially traced to different C–H vs O–H bond strengths. Indeed, we found that the total bond-dissociation energy required to liberate the two migrating hydrogen atoms is approximately 7 kcal/mol lower in CH_3_–OH than in CH_3_–CH_3_.

**Figure 6:**
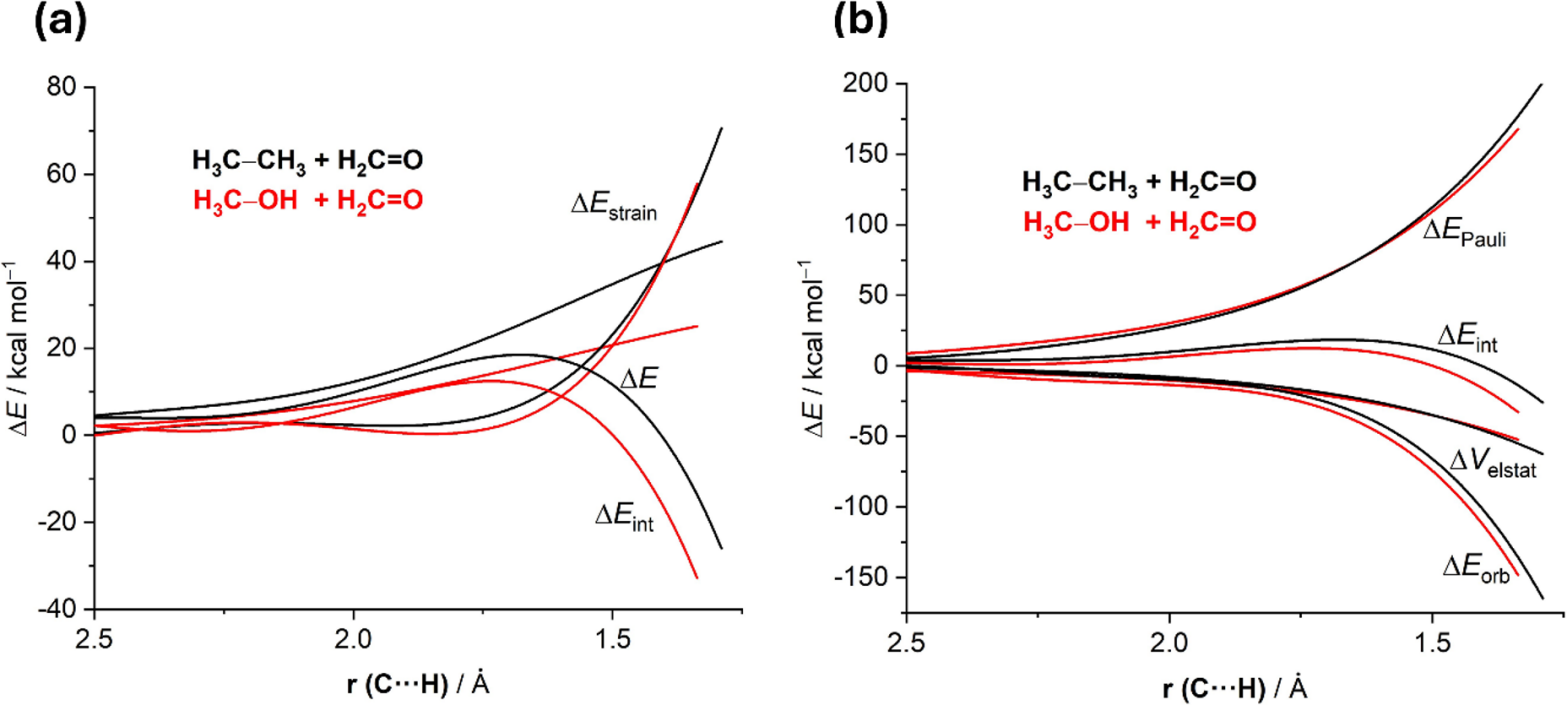
Comparative activation strain analyses (a) and energy decomposition analysis (b) of the DGRT between ethane and formaldehyde (black lines) and the analogous process involving methanol (red lines) and projected onto the C···H bond-forming distance.

More quantitative insights into the factors leading to the barrier reduction were gained by applying the combined ASM-EDA method [79]. As graphically shown in Figure 6a, which compares the corresponding ASDs for both DGTRs from the beginning of the process up to the respective (aromatic) transition states, it becomes clear that the lower barrier computed for the process involving CH_3_–OH derives solely from the stronger interaction between the deformed reactants along the entire reaction coordinate, and particularly, at the transition state region. This results from the formation of a stabilizing, intramolecular CH_3_–OH···O=CH_2_ hydrogen bond at the initial stages of the process which brings together both reactants, significantly enhancing the orbital interactions (mainly HOMO–LUMO) between them (as confirmed by the EDA method, Figure 6b). Obviously, this stabilizing noncovalent interaction is lacking in the process involving ethane as hydrogen donor and as a consequence, the activation barrier computed for this DGTR is substantially higher (although the in-plane aromaticity of the corresponding transition states is comparable).

#### Thermal cycloisomerization of 1,3-hexadien-5-ynes (Hopf cyclization)

**Scheme 2:**
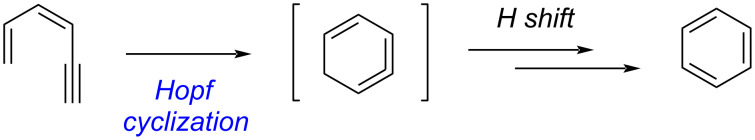
Representative cycloisomerization reaction of 1,3-hexadien-5-yne.

Since the original report by Hopf and Musso in 1969 [80], the thermal cycloisomerization reactions of 1,3-haxedien-5-ynes have been widely applied to the synthesis of aromatic six-membered rings [81]. From a mechanistic point of view, this process involves the initial formation of a bent-allene intermediate, which leads to the final reaction product via hydrogen shifts (Scheme 2) [82].

**Figure 7:**
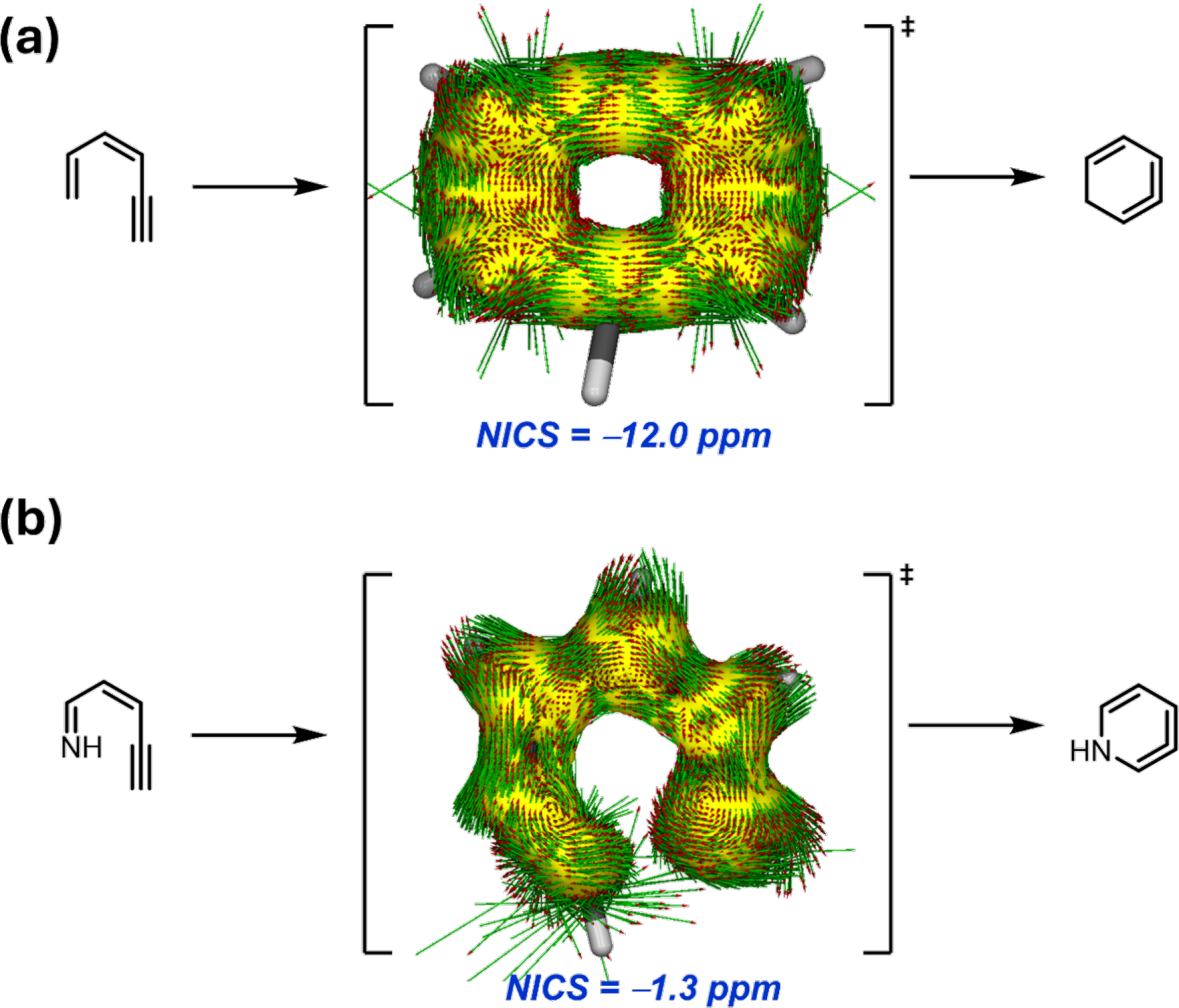
AICD plots of the transition states associated with the Hopf cyclization reactions involving *cis*-hexa-1,3-diene-5-yne (a) and the analogous process involving its imine counterpart (b).

The so-called Bergman cyclization of *cis*-3-hexene-1,5-diynes [83–84], which is suggested to proceed through an in-plane aromatic transition state, is analogous to the first step of this transformation, known as Hopf cyclization. Indeed, our calculations [85] indicate that the parent Hopf cyclization involving *cis*-hexa-1,3-diene-5-yne occurs in a concerted manner through a transition state that features in-plane aromaticity in view of the computed NICS(3, +1) value of −12.0 ppm and diatropic induced ring current (Figure 7a). Interestingly, the analogous reaction involving *cis*-pent-2-en-4-yn-1-imine, where the terminal CH=CH_2_ group in the parent system was replaced by an imine CH=NH group, proceeds with a much lower barrier (Δ*E*^≠^ = 23.8 kcal/mol vs 36.8 kcal/mol, respectively). However, the corresponding six-membered transition state is essentially non-aromatic according to the computed NICS(3, +1) value of −1.3 ppm, which results from the interruption of the six-electron delocalization (Figure 7b). A similar behavior was observed in the analogous aldehyde counterpart (*cis*-pent-2-en-4-ynal) whose cyclization proceeds with a lower barrier than the parent system (Δ*E*^≠^ = 30.3 kcal/mol) although the corresponding transition state is also non-aromatic (NICS(3, +1) = −1.5 ppm. Therefore, we found once again that the gain in (in-plane) aromaticity in the parent reaction is not translated into a gain in stability (i.e., a more aromatic transition structure does not translate into a lower-barrier process).

**Figure 8:**
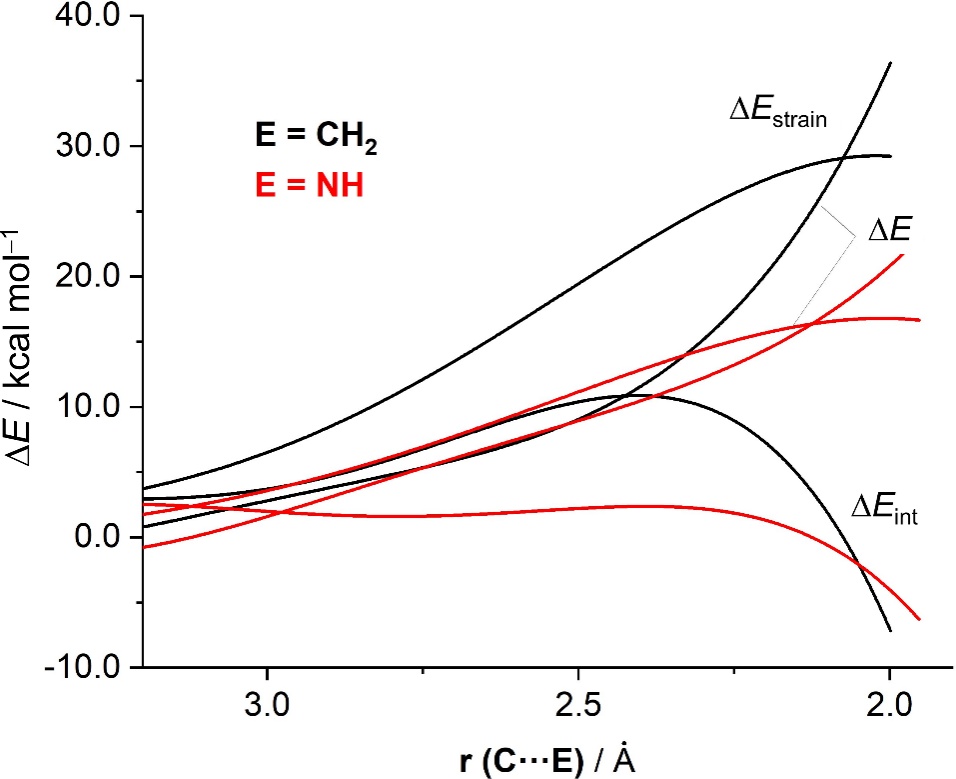
Comparative activation strain analyses of the Hopf cyclization involving ene–ene–ynes E=CH–CH=CH–C≡CH (E = CH_2_, black lines; E = NH, red lines) ethane and formaldehyde (black lines) and projected onto the C···E bond-forming distance.

Following the same methodology, the ASM of reactivity was applied then to understand the reasons behind the lower barriers computed for the Hopf cyclizations of ene–ene–ynes E=CH–CH=CH–C≡CH where the terminal CH_2_ group was replaced by a heteroatom (E = NH or O). Figure 8 shows the corresponding ASDs for the parent cyclization (E = CH_2_) and the analogous process involving the imine system (E = NH). In this case, as the reactions are intramolecular, the ASM terms are referred to the initial reactants and are computed by considering the interaction between E=CH^•^ and ^•^CH=CH–C≡CH radicals. As shown in Figure 8, although the change in the interaction (ΔΔ*E*_int_) between the fragments is stronger for the imine system at the initial stages of the cyclization, this term becomes rather similar for both systems at the proximity of the corresponding transition states and therefore, is not responsible for the different barriers. Instead, the cyclization involving the parent system is associated with a much more destabilizing strain energy. This derives from the rotation of the CH_2_ group required to maximize the π-HOMO(CH=CH_2_)/π*-LUMO(C≡CH) interaction. As the HOMO of the imine system is located on the lone pair (LP) of the nitrogen atom, such rotation is no longer required, which translates into the computed lower strain energy and ultimately into the lower barrier.

#### 1,3-Dipolar cycloaddition reactions between azides and metal cyaphide complexes

Similar to the Diels–Alder cycloaddition reaction, the 1,3-dipolar cycloaddition between a 1,3-dipole (acting as 4π system) and a 2π dipolarophile is a widely used transformation in synthesis, typically leading to five-membered heterocycles [86–87]. Although introduced by Huisgen in 1960 [88–90], this process has experienced a new renaissance in the last decades due to its countless applications in different fields, and particularly in bioorthogonal chemistry [91–93].

**Scheme 3:**
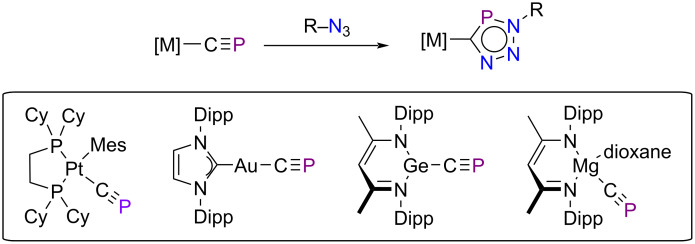
1,3-Dipolar cycloaddition reactions between *t-*BuN_3_ and cyaphide complexes.

Among the number of dipolarophiles compatible with this [3 + 2]-cycloaddition, phosphaalkynes [94–96] and arsaalkynes [97] have been recently used because they allow easy access to novel heterocycles. For instance, when using organic azides as dipoles, the transformation leads to the preparation of triazaphospholes and triazaarsoles, π-conjugated species with potential applications in materials science due to their luminescent properties [98]. However, these heavier alkyne analogues are typically kinetically unstable, which limits their use as dipolarophiles. Despite that, very recently it was found that the C≡P moiety, in particular, can be stabilized in the form of a cyaphide ligand bonded to a metal fragment [99–100]. These cyaphide complexes are proven to readily undergo 1,3-dipolar cycloaddition reactions with organic azides [99–101], affording novel metal triazaphospholes (Scheme 3) which can be further transformed into protio- and iodotriazaphospholes [101].

**Figure 9:**
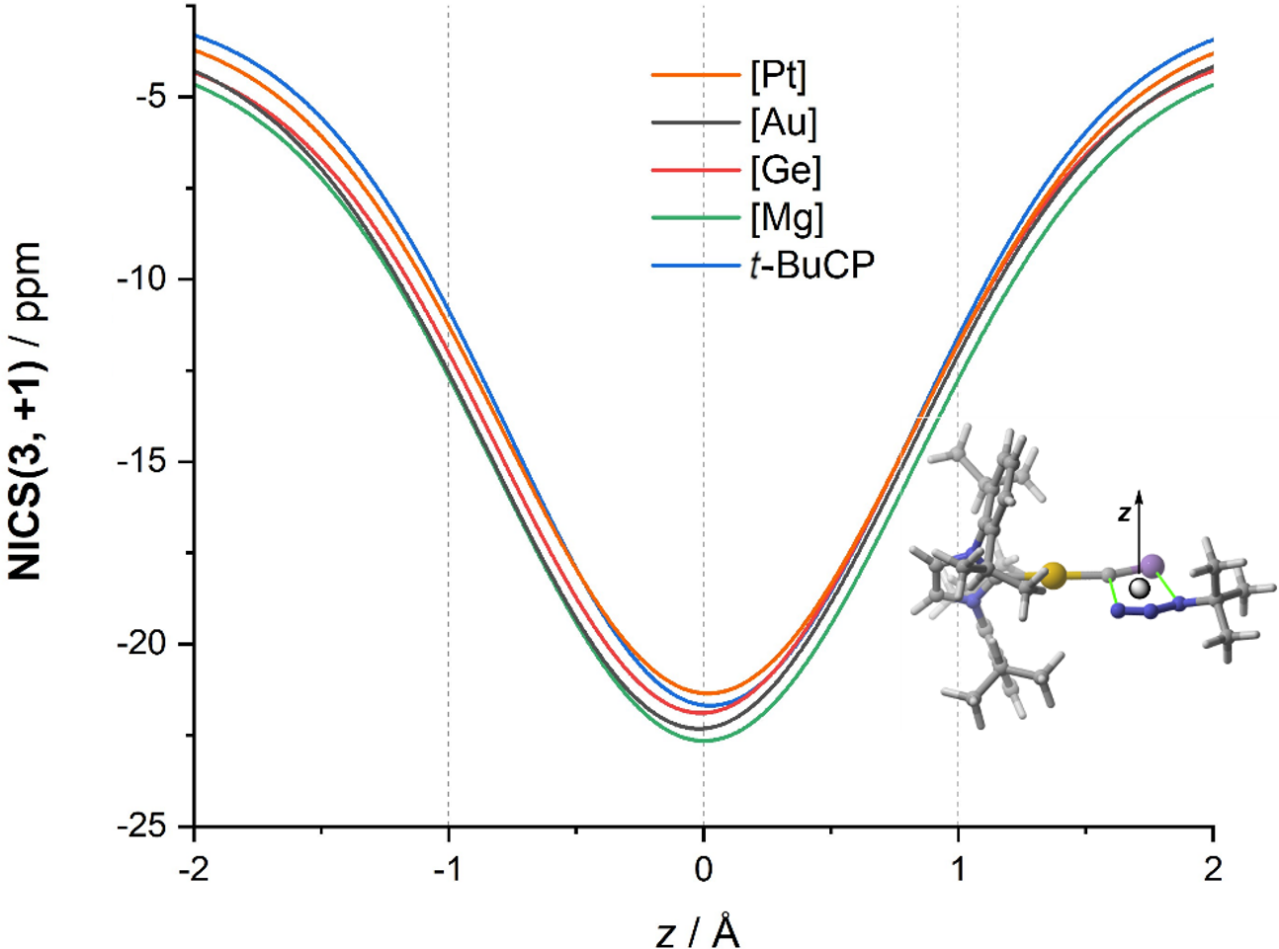
Evolution of the NICS(3, +1) values along a *z*-axis perpendicular to the molecular plane of the TSs involved in the 1,3-dipolar cycloaddition reactions between *t-*BuN_3_ and cyaphide complexes.

We first compared these metal cyaphide/azide cycloadditions with the analogous non-metallic process involving *t-*BuC≡P as dipolarophile [102–103]. Interestingly, we found that, although in all cases the process occurs in a concerted manner through the corresponding five-membered transition state, the germanium and magnesium cyaphide complexes are significantly more reactive (i.e., leading to a lower-barrier process, Δ*G*^≠^ ≈ 20 kcal/mol) than their platinum or gold counterparts, which exhibit barriers comparable to that computed for the non-metallic process (Δ*G*^≠^ ≈ 26 kcal/mol). Once again, we explored the impact of the aromaticity of the involved transition structures on the computed barriers. As related [3 + 2] cycloaddition reactions [22,45,104–105], the transition states associated with these cycloadditions involving metal cyaphides should be in-plane aromatic species due to the cyclic delocalization of 6-electrons in the molecular plane. Indeed, the evolution of the NICS(3, +1) values along a *z*-axis perpendicular to the molecular plane shows the expected bell shape with a maximum value at *z* = 0 Å (Figure 9). Despite that, the computed NICS(3, +1) values are rather similar for all the considered transition structures (ca. −25 ppm), which suggests that, once again, there is no clear relationship between in-plane aromaticity and reactivity.

**Figure 10:**
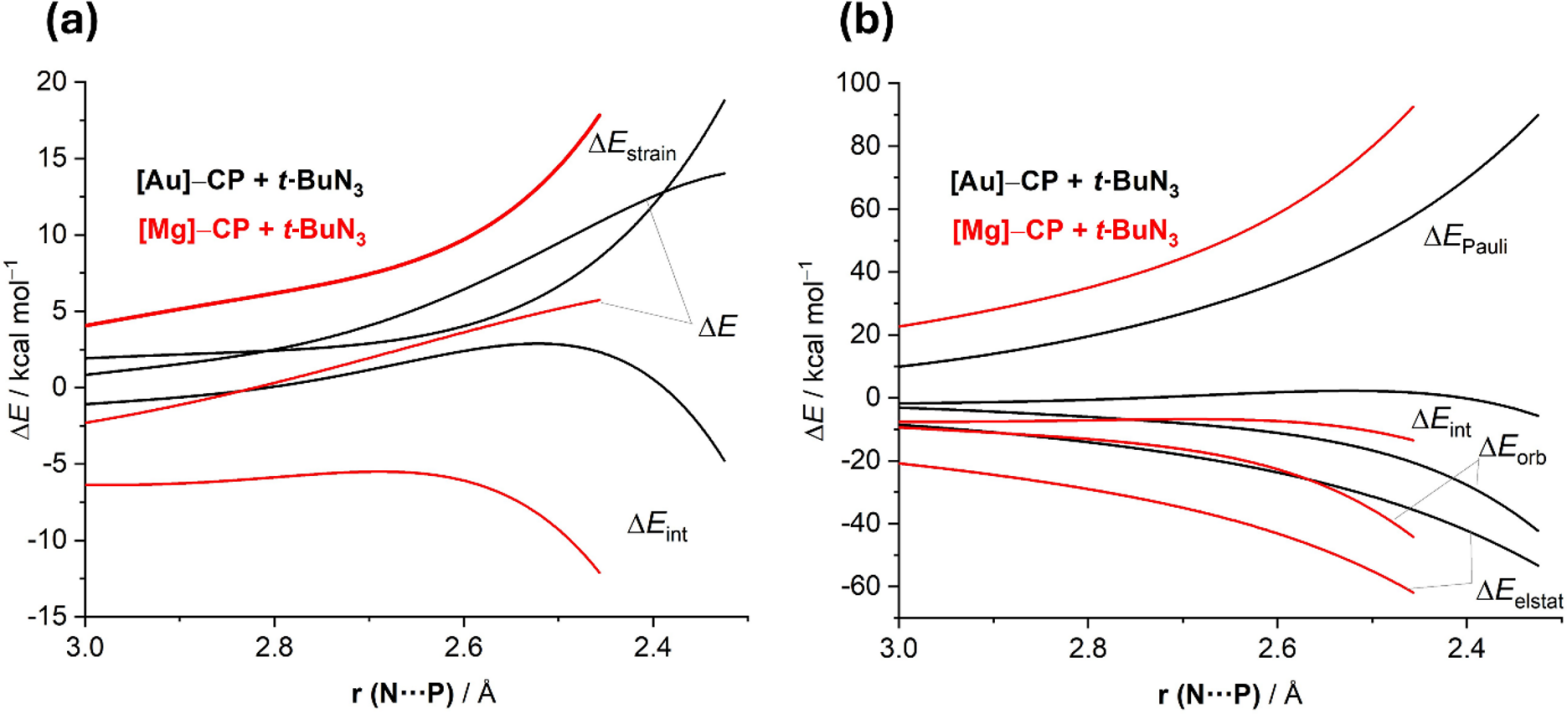
Comparative activation strain analyses (a) and energy decomposition analysis (b) of the 1,3-dipolar cycloaddition reactions between *t-*BuN_3_ and [Au]CP complex (black lines) and the analogous process involving [Mg]CP complex and projected onto the N···P bond-forming distance.

To understand the factors leading to the lower barriers computed for the 1,3-dipolar cycloadditions involving Mg and Ge cyaphide complexes, the ASM of reactivity was applied. Figure 10a shows the ASDs for the [3 + 2]-cycloaddition reactions between *t-*BuN_3_ and [Au]C≡P and [Mg]C≡P cyaphide complexes, as representative systems, from the initial stages of the process up to the corresponding transition states. It is found that the lower barrier computed for the cycloaddition involving the Mg cyaphide complex does not originate from the strain energy, which is actually much less destabilizing for the analogous reaction involving the gold(I)–cyaphide complex. Instead, the former reaction benefits from a much stronger interaction between the deformed reactants along the entire reaction coordinate, which compensates for the destabilization provoked by the significant deformation required in this transformation. According to the EDA method, this stronger interaction derives from both electrostatic and orbital interactions, which are markedly more stabilizing for the process involving the magnesium cyaphide complex. The stronger electrostatic interactions mainly result from the significant polarization of the cyaphide ligand induced by the highly electropositive magnesium atom whereas the stronger orbital interactions derive from a more stabilizing direct π(C≡P)→π*(azide) molecular orbital interaction coupled with a stronger reverse π(azide)→π*(C≡P) interaction.

## Conclusion

Through selected representative examples, in this perspective article the interplay between transition-state aromaticity and reactivity in concerted pericyclic reactions has been discussed. Not surprisingly, the strength of the aromaticity of the transition states strongly depends on their geometry in the sense that a higher degree of synchronicity is typically associated with a more aromatic species. Although aromaticity and energetic stabilization are traditionally connected in stable species (i.e., minima on the potential energy surface), it is found that processes featuring more asynchronous (less aromatic) transition states exhibit lower barriers than their more synchronous counterparts. This finding, which is systematically found regardless of the nature of the pericyclic reaction (from catalyzed Diels–Alder cycloaddition reactions to uncatalyzed double group transfer reactions) therefore indicates that the influence of the TS-aromaticity on the barrier heights of these transformations is not significant (or even, negligible). Instead, other factors such as the reduction of the Pauli repulsion or more favorable electrostatic/orbital interactions, as revealed by the ASM-EDA method, become the ultimate factors controlling the barriers of these reactions.

## Data Availability

Data sharing is not applicable as no new data was generated or analyzed in this study.
